# Distinct Cortical Microstructure in Postmenopausal Women with Distal Radius and Proximal Humerus Fractures

**DOI:** 10.1007/s00223-026-01579-7

**Published:** 2026-07-21

**Authors:** Mikolaj Bartosik, Alexander Simon, Oskar Windels, Felix N. von Brackel, Florian Barvencik, Michael Amling, Ralf Oheim

**Affiliations:** Department of Osteology and Biomechanics, University Medical Center Hamburg-Eppendorf, Lottestrasse 59, 22529 Hamburg, Germany

**Keywords:** Postmenopausal osteoporosis, Fragility fractures, Radius fracture, Humerus fracture, High resolution peripheral quantitative computer tomography

## Abstract

Distal radius fractures and proximal humerus fractures are common fractures in postmenopausal women with osteoporosis. The question of whether bone microstructural deficits are reflected in specific changes in bone microstructure at peripheral skeletal sites has not yet been investigated. In this retrospective study, treatment-naïve postmenopausal women with distal radius fractures (DRF) or proximal humerus fractures (PHF) were included. Of more than 500 patients screened, 30 patients with DRF and 30 patients with PHF were included in the final analysis following propensity score matching. All participants underwent biochemical testing, dual-energy X-ray absorptiometry assessment, and bone microstructural evaluation by high-resolution peripheral quantitative computed tomography. After propensity score matching, both groups showed no significant differences in terms of age, weight, height, BMI, and lowest T-score (all *p* > 0.05). Mean age was 64.4 ± 10.7 years in the DRF group and 66.7 ± 9.0 years in the PHF group (*p* = 0.365). In both groups, the lowest mean T-score was − 2.9 (*p* = 0.539). Overall, both groups showed a reduction in total BMD and microarchitectural parameters compared to reference values at the distal radius and tibia. The PHF group exhibited significantly reduced bone geometry at the distal radius compared with the DRF group, including lower total area (p = 0.004), trabecular area (*p* = 0.021), and cortical area (*p* = 0.006), as well as reduced cortical thickness at both the distal radius (*p* = 0.031) and tibia (*p* = 0.024). PHF are characterized by reduced cortical thickness and unfavorable bone geometry, which is independent of areal bone mineral density.

**Supplementary Information:**

The online version contains supplementary material available at 10.1007/s00223-026-01579-7.

## Introduction

Fragility fractures represent a major public health burden among postmenopausal women and are associated with substantial morbidity, loss of independence, and increased mortality [1–3]. Along with vertebral fractures and proximal femur fractures, distal radius fractures and proximal humerus fractures are among the most common manifestations of osteoporotic fractures [4–6] and are considered as major osteoporotic fractures. While areal bone mineral density (aBMD) assessed by dual-energy X-ray absorptiometry (DXA) remains the clinical standard for fracture risk assessment, a large proportion of fractures occur in individuals who do not meet densitometric criteria for osteoporosis [7, 8]. This observation underscores the importance of skeletal properties beyond bone mass, including bone geometry and microarchitecture, in determining bone strength and fracture susceptibility [9–12]. Whether different fragility fracture phenotypes reflect distinct systemic skeletal microstructural patterns remains incompletely understood, particularly with regard to fractures occurring at different anatomical sites.

Low traumatic distal radius fractures and proximal humerus fractures are common peripheral fragility fractures in postmenopausal women and are considered indicators of skeletal fragility [13, 14]. Despite their shared clinical context of fragility fractures, both differ in terms of trauma mechanism, anatomical composition, and potential underlying bone deficits. Whether these differences are reflected in site-specific alterations of bone microarchitecture at peripheral skeletal sites has not been systematically investigated.

In the present study, we investigated possible differences in peripheral bone architecture between treatment-naïve postmenopausal women with osteoporosis and recent distal radius fractures (DRF) compared to those with recent proximal humerus fractures (PHF). By comparing these two common fragility fracture entities in propensity score-matched cohorts, we sought to explore microstructural and geometric characteristics that may be associated with fracture susceptibility beyond aBMD.

## Methods

### Study Design

This retrospective study was conducted in accordance with the Declaration of Helsinki as well as local ethical guidelines. Treatment-naïve postmenopausal women presenting to our outpatient clinic with a recently experienced a PHF or DRF were considered for inclusion. Patients with a PHF or DRF were eligible if the fracture had occurred within 24 months to the timepoint of measurement.

Patients were excluded if they had bone-related tumors, hereditary bone diseases or secondary osteoporosis such as systemic glucocorticoid-induced osteoporosis, as well as fractures resulting from high energy or adequate trauma. To reduce important confounding factors age and lowest T-score, propensity score matching was performed. All participants underwent biochemical analyses, areal bone mineral density assessment using dual-energy X-ray absorptiometry (DXA), and bone microstructural evaluation using high-resolution peripheral quantitative computed tomography (HR-pQCT) as part of routine clinical care. Data on peripheral and vertebral fractures were collected using standardized medical records. Vertebral fractures were additionally detected by lateral vertebral assessment in patients with a corresponding indication, such as significant height loss or spinal pain in physical examination, which was present in 93% of cases.

### Biochemical Analysis

We routinely processed blood and urine samples at the local laboratory using standardized laboratory protocols with internal quality controls. Concentrations of serum calcium, serum phosphate, 25-hydroxyvitamin D (25(OH)D), parathyroid hormone (PTH), alkaline phosphatase (ALP), osteocalcin, bone-specific alkaline phosphatase (b-ALP), N-terminal propeptide of type I procollagen (P1NP), C-terminal telopeptide of type I collagen (CTX) and urinary deoxypyridinoline-to-creatinine (DPD) ratios were determined.

### Dual-energy X-ray Absorptiometry (DXA)

To assess bone mineral density patients received dual-energy X-ray absorptiometry (Lunar iDXA, GE Healthcare, Madison, Wisconsin, USA) examinations on the lumbar spine (L1-4) and proximal femurs with measurements of femoral neck and total hip. The lowest T-score was then determined with the corresponding aBMD, irrespective of skeletal site (lumbar spine, femoral neck, or total hip). Daily quality control of the DXA devices was conducted through calibration scans using a standard phantom, following the manufacturer’s instructions. Precision evaluation also involved determining the least significant change in line with the guidelines of the International Society for Clinical Densitometry (ISCD).

### High Resolution Peripheral Quantitative Computed Tomography (HR-pQCT)

Patients received either first- (XCT1) or second-generation (XCT2) HR-pQCT (XtremeCT and XtremeCT II, Scanco Medical AG, Brüttisellen, Switzerland) measurements of the non-dominant distal radius and the opposite distal tibia, according to the manufacturer's standard in vivo scanning protocol for the respective HR-pQCT, previously described elsewhere [15]. In cases of a fracture affecting the non-dominant arm, measurements were performed on the contralateral side instead. Microarchitecture parameters followed the standardized nomenclature of the IOF-ASBMR-ECTS working group [16]. In the PHF group, 14 of 30 patients (47%) were scanned using XCT1 and the remaining 16 (53%) using XCT2, whereas all patients in the DRF group (30 of 30, 100%) were scanned using XCT2. A similar pattern was observed when restricting the analysis to XCT2 data only. In order to enable comparability between devices, sex and age groups, HR-pQCT parameters were adjusted for normality, and the percentage median (% median) was calculated based on device-, age-, and sex-specific reference values for subsequent analyses [17, 18]. Mean and borders of the used reference groups were converted according to Manske et al. [19] to ensure the comparability of the reference groups used for first- and second-generation devices and to prevent bias when using median values.

### Muscle and Balance Performance

Muscle and balance performance parameters were assessed using the Leonardo Mechanograph, Novotec Medical, Pforzheim, Germany, as described elsewhere [20]. Maximum grip strength in kilograms was measured using a hand held dynamometer Leonardo Mechanograph GF. Chair rise test (CRT) time per repetition in seconds was recorded using a ground reaction force plate (GRFP) Leonardo Mechanograph GRFP STD. Postural stability was evaluated with the Leonardo Mechanograph GRFP STD by measuring Romberg center of pressure movements, expressed as path length in millimeters, under eyes open and eyes closed conditions for 10 s.

### Statistical Analysis

For statistical analysis, SPSS Statistics 31.0.0 (IBM, Armonk, New York, USA), JASP 0.19.1 (University of Amsterdam, Netherlands), JASP 0.19.1 (University of Amsterdam, Netherlands) and GraphPad Prism 10.4.1 (GraphPad Software, San Diego, California, USA) were used. After the initial preselection, propensity score matching of age and lowest T-score was performed to improve comparability. The results were reported as mean ± standard deviation (SD) and % median of the reference values for the HR-pQCT parameters. The Shapiro–Wilk test was used to assess the normal distribution of the data. Comparisons between two subgroups were performed using an unpaired two-tailed t-test for parametric data and the Mann–Whitney U test for nonparametric data. Effect size is reported as Cohen’s d. The Fisher´s exact test was used to compare all categorical variables. Correlation analyses were performed to examine associations of fracture type and frequency with bone microstructure and density parameters measured by HR-pQCT.

## Results

### Characterization of the Study Cohort

The characteristics of our propensity score matched study cohort are presented in Table 1. More than 500 patients were screened for eligibility. Of these, 66 patients met the inclusion criteria for recent DRF and 87 patients met the inclusion criteria for recent PHF. Following 1:1 propensity score matching for age and lowest T-score, 30 patients per group were included in the final analysis, comprising 60 patients in total. After propensity score matching for age and lowest T-score the group comparisons between patients with DRF or PHF showed no significant differences in age, height, weight, BMI as well as lowest T-score (all *p* > 0.05). The mean age was 64.4 ± 10.7 years in the DRF group and 66.7 ± 9.0 years in the PHF group (*p* = 0.365, d = 0.23). The lowest T-score was − 2.9 in both groups (*p* = 0.539, d = 0.16). Furthermore, region-specific T-scores showed a similar pattern between groups at the femur (DRF: − 2.6 ± 0.6; PHF: − 2.4 ± 0.9; *p* = 0.196, d = 0.26) and at the spine (DRF: -2.2 ± 1.2; PHF: − 2.8 ± 1.3; *p* = 0.087, d = 0.46). One patient in the DRF group (1 of 30, 3%) had a diagnosis of diabetes mellitus type 1, whereas no patient in the PHF group had diabetes. Obesity (BMI > 25 kg/m^2^) was present in 8 of 30 patients (27%) in the PHF group and 7 of 30 patients (23%) in the DRF group (p > 0.999). Overall comorbidity burden, quantified using the Charlson Comorbidity Index (CCI), did not differ significantly between groups (0.10 ± 0.30 in DRF vs. 0.27 ± 0.63 in PHF; *p* = 0.394). A significant difference was observed in the number of peripheral fragility fractures, with the DRF group exhibiting a higher mean number of peripheral fractures (2.1 ± 1.3) compared with the PHF group (0.8 ± 1.0; *p* < 0.001, d = 1.21). No significant difference was found in the frequency of vertebral fragility fractures.

**Table 1:** Overview of the total cohort of postmenopausal patients categorized by distal radius fracture (DRF) and proximal humerus fracture (PHF).

	DRF (n = 30)				PHF (n = 30)					
Parameter	Mean	SD	Min	Max	Mean	SD	Min	Max	p-value	d
*Demographics*										
Age (years)	64.4	10.7	40.0	95.0	66.7	9.0	52.0	89.0	0.365	0.23
Weight (kg)	60.1	11.3	41.0	85.0	63.9	13.7	41.7	118.0	0.735	0.29
Height (m)	1.65	0.06	1.54	1.77	1.64	0.08	1.50	1.87	0.270	0.09
BMI (kg/m^2^)	22.2	4.0	15.0	33.4	23.7	4.7	16.7	41.8	0.165	0.37
*DXA*										
Femoral T-score	− 2.6	0.6	− 4.1	− 1.4	− 2.4	0.9	− 4.4	− 0.9	0.196	0.26
Femoral aBMD	0.667	0.074	0.483	0.812	0.701	0.111	0.404	0.867	0.179	0.36
Spinal T-score	− 2.2	1.2	− 5.4	0.2	− 2.8	1.3	− 5.9	1.0	0.087	0.46
Spinal aBMD	0.885	0.129	0.522	1.230	0.838	0.164	0.456	1.280	0.234	0.32
Lowest T-score	− 2.9	0.7	− 5.4	− 1.0	− 2.9	1.3	− 5.9	1.0	0.539	0.16
Lowest aBMD	0.690	0.103	0.483	0.892	0.761	0.175	0.404	1.283	0.060	0.50
*Fragility fractures*										
Vertebral fractures	0.7	1.5	0.0	6.0	1.6	2.4	0.0	10.0	0.060	0.44
Peripheral fractures	2.1	1.3	1.0	6.0	0.8	1.0	0.0	4.0	** < 0.001**	**1.21**

Fragility fractures were further classified into vertebral and peripheral fractures with mean values provided. SD standard deviation, BMI body mass index, DXA dual-energy X-ray absorptiometry. Numbers in bold indicate statistical significance (*p* < 0.05). Effect sizes are given as Cohen's d, with strong effect sizes (Cohen's d > 0.8) highlighted in bold

### Laboratory Parameters in Patients with DRF and PHF

Among the laboratory parameters related to calcium and bone metabolism, the only significant difference was observed in serum 25(OH)D levels (Supplementary Table 1). But both groups exhibited sufficient 25(OH)D concentrations (≥ 30.0 µg/L), the DRF group showed higher mean levels compared with the PHF group (39.1 ± 13.9 µg/L in DRF vs. 32.8 ± 13.6 µg/L in PHF; *p* = 0.042, d = 0.56). Furthermore, both groups demonstrated increased bone resorption, as reflected by elevated DPD levels above the reference range of 2–7 nmol/mmol (9 ± 3 nmol/mmol in DRF vs. 9 ± 2 nmol/mmol in PHF; *p* = 0.835, d = 0.06). No additional significant differences were identified across the remaining laboratory parameters (Supplementary Table 1).

### Comparison of Bone Microarchitecture Using HR-pQCT Parameters

**Fig. 1:**
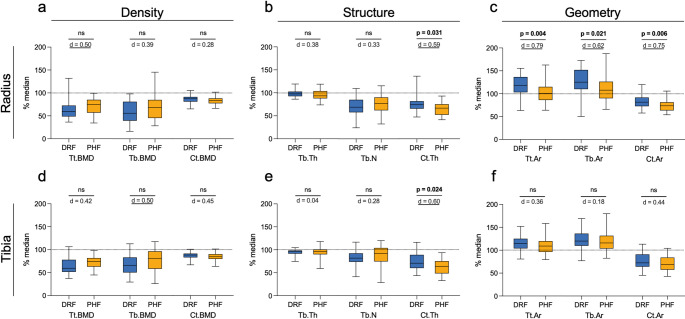
Differences in bone microarchitecture according to distal radius fracture (DRF) and proximal humerus fracture (PHF). Patients with PHF showed reduced cortical thickness (Ct.Th) at both the distal radius (b) and tibia (e), as well as reduced bone geometry at the radius (c), compared with DRF patients. HR-pQCT values are expressed as a percentage of device-, age-, and sex-specific median reference values (XCT1: [17]; XCT2: [18]). Abbreviations: Tt.BMD, total BMD; Tb.BMD, trabecular BMD; Ct.BMD, cortical BMD; Tb.Th, trabecular thickness; Tb.N, trabecular number; Ct.Th, cortical thickness; Tt.Ar, total area; Tb.Ar, trabecular area; Ct.Ar, cortical area. Exact p-values show significant group differences. Medium effect sizes (Cohen's d > 0.5) are underlined

Relative to the reference data, the overall cohort exhibited markedly reduced total, trabecular and cortical volumetric BMD, deterioration of both trabecular and cortical microarchitecture, and reduced cortical area at both distal radius and tibia. Comparison of HR-pQCT parameters between the DRF and PHF groups revealed significant differences, particularly in radial bone geometry with moderate effect sizes (Fig. 1). At the distal radius, the PHF group exhibited significantly reduced total area (Tt.Ar), trabecular area (Tb.Ar), and cortical area (Ct.Ar) compared with the DRF group (Tt.Ar: 119.0 ± 21.3% median in DRF vs. 102.0 ± 22.0% median in PHF, *p* = 0.004, d = 0.79; Tb.Ar: 127.0 ± 27.9% median in DRF vs. 110.0 ± 27.4% median in PHF, *p* = 0.021, d = 0.62; Ct.Ar: 84.3 ± 15.2% median in DRF vs. 73.7 ± 12.8% median in PHF, *p* = 0.006, d = 0.75) (Fig. 1c). In addition, the cortical structure of PHF patients was significantly reduced compared to DRF patients, with cortical thickness (Ct.Th) being significantly lower in the PHF group at both the distal radius (Fig. 1b) and tibia (Fig. 1e) (radius: 77.5 ± 20.2% median in DRF vs. 66.0 ± 14.2% median in PHF, *p* = 0.031, d = 0.59; tibia: 73.9 ± 18.9% median in DRF vs. 63.0 ± 17.1% median in PHF, *p* = 0.024, d = 0.60).

No further significant differences were observed between the two groups in terms of bone density or the other trabecular and cortical parameters. Noteworthily both groups demonstrated reduced density and trabecular and cortical architecture (Fig. 1a–b, Fig. 1d–e).

### Correlation Analyses of Fracture Frequency and Bone Microstructure Parameters According to HR-pQCT

**Fig. 2:**
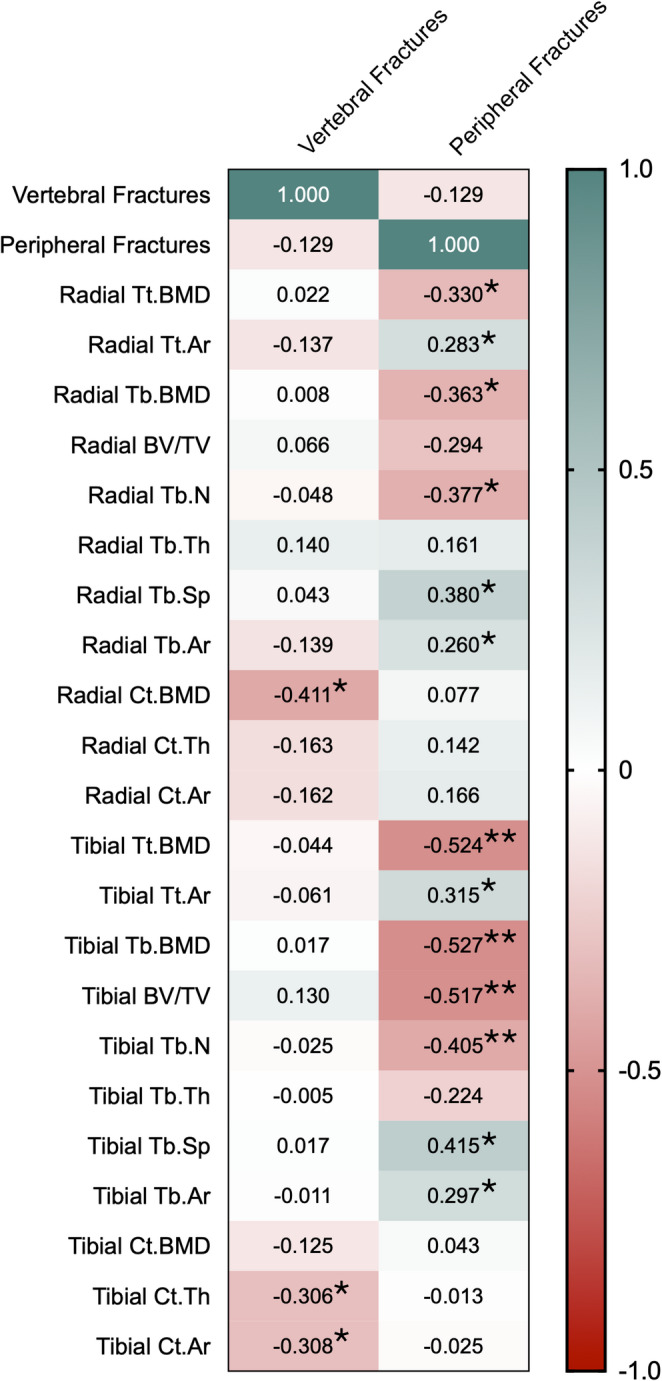
Correlation analyses in the overall cohort of vertebral and peripheral fragility fracture frequency and bone microstructure parameters. Vertebral fracture number correlated negatively with radial cortical density, structure, and geometry. Peripheral fracture number correlated with impaired trabecular density and structure at the tibia. Abbreviations: Tt.BMD, total BMD; Ct.BMD, cortical BMD; Tb.BMD, trabecular BMD; BV/TV, bone volume/total volume; Tb.N, trabecular number; Tb.Th, trabecular thickness; Tb.Sp, trabecular separation; Ct.Th, cortical thickness; Tt.Ar, total area; Tb.Ar, trabecular area; Ct.Ar, cortical area. §, % median; *p < 0.05; **p < 0.001

To assess the relationship between fragility fracture frequency and bone microstructure, correlation analyses were performed across the overall cohort. Significant negative correlations were observed between the number of vertebral fractures and radial cortical bone mineral density (Ct.BMD; r_s_ = − 0.411), as well as tibial cortical thickness (Ct.Th; r_s_ = − 0.306) and cortical area (Ct.Ar; r_s_ = − 0.308) (Fig. 2).

With regard to peripheral fragility fractures, impaired trabecular bone mineral density and microstructural parameters in both the radius and tibia were significantly associated with the number of fractures (Fig. 2). The strongest significant correlations were observed between peripheral fractures and Tb.BMD in the radius (r_s_ = -0.363) and tibia (r_s_ = -0.527).

### Comparison of Muscle and Balance Performance

When comparing the muscle and balance performance parameters, we found no significant differences in grip strength (19.0 ± 7.6 kg in DRF vs. 16.0 ± 8.6 kg in PHF, *p* = 0.225, d = 0.45), CRT time per repetition (1.79 ± 0.59 s in DRF vs. 2.15 ± 1.38 s in PHF, *p* = 0.456, d = 0.46), or Romberg path length with eyes open (157.6 ± 67.2 mm in DRF vs. 151.6 ± 56.6 mm in PHF, *p* > 0.999, d < 0.01) or closed conditions. However, for Romberg path length with eyes closed, the DRF cohort showed a greater path length (276.0 ± 131.9 mm) compared with the PHF cohort (229.3 ± 188.9 mm), with a moderate effect size but without reaching statistical significance (p = 0.113, d = 0.59) (Supplementary Table 2).

## Discussion

Distal radius fractures and proximal humerus fractures are classified as major osteoporotic fractures and represent key indicators of underlying skeletal fragility [21, 22]. Despite being grouped in the category of major osteoporotic fractures, distal radius and proximal humerus fractures differ substantially with respect to injury mechanism. Nevertheless, both fracture types share common risk factors, including reduced bone density and an increased risk of falling. These observations suggest that, beyond aBMD, bone microarchitecture may contribute to fracture susceptibility and localization [10, 23]. This study investigated microstructural differences between patients with DRF and PHF, controlling for key clinical confounders, using propensity score matching.

Along with low bone density, bone microstructural alteration is a major risk factor for fragility fractures [10, 24, 25]. Despite no difference in lowest T-scores, both groups demonstrated reductions in Tt.BMD and microarchitectural parameters at the distal radius and tibia. Reduced bone architecture is associated with an increased risk of fragility fractures [26, 27], even in women with normal to slightly reduced aBMD [10, 24, 28]. These findings support the potential value of structural phenotyping to improve fracture risk stratification beyond conventional aBMD assessment [26, 29]. Therefore, a detailed assessment of both bone density and microarchitecture could be used in the future for fracture prediction [30] and risk stratification, as well as for individualized prevention strategies [31]. Particularly noteworthy is the additional significant reduction in Tt.Ar, Tb.Ar and Ct.Ar at the distal radius in the PHF cohort. Even small reductions in cross-sectional bone area may substantially reduce bending strength due to the relationship between bone perimeter and load-bearing capacity [32–34]. A smaller bone area therefore implies a reduced load-bearing capacity, even with comparable volumetric or aBMD.

In both cohorts, Ct.Th was reduced at the distal radius and distal tibia, but patients with PHF exhibited significantly lower Ct.Th at both sites compared with those sustaining DRF. The observed microstructural deficits suggest that cortical thinning and reduced bone area may be associated with increased PHF susceptibility. Our results suggest that patients with PHF may exhibit distinct deficits in cortical thickness and bone geometry compared to patients with DRF, despite similar aBMD. These site-specific microstructural differences suggest that fracture localization may be influenced by subtle variations in cortical geometry and bone strength, highlighting heterogeneity in skeletal fragility. Consistent with these findings, Yu et al. demonstrated that even anatomically adjoining fracture types, such as femoral neck and subtrochanteric fractures, exhibit distinct cortical microarchitectural patterns (measured by QCT) [35]. While both fracture types showed thinner cortices compared with controls, femoral neck fractures were characterized by more focal cortical thinning. Such observations support the concept that subtle, site-specific variations in bone geometry and microstructure may differentiate fracture phenotypes, reinforcing the relevance of microarchitectural assessment in understanding fracture susceptibility [36, 37].

Interestingly, despite exhibiting comparatively greater cortical thickness and larger bone area, the DRF cohort sustained a higher number of peripheral fractures than the PHF cohort. This suggests that factors beyond bone microstructure, such as subtle impairments in postural control and the associated increased risk of falling, may contribute to fracture risk in the DRF group. Although muscle and balance performance parameters did not differ significantly between both groups, the DRF cohort showed a tendency toward greater Romberg path length with eyes closed, with a moderate effect size. These findings underscore the multifactorial nature of fragility fractures, reflecting the combined influence of skeletal and fall related risk factors [38–40]. This discrepancy between microarchitectural findings and observed fracture frequency illustrates that the HR-pQCT differences reported here should not be interpreted as a direct measure of overall fracture risk, but rather as evidence of distinct skeletal phenotypes that may interact with non-skeletal factors, such as fall mechanics and frailty, in determining fracture location.

In an exploratory analysis, we assessed the relationship between microstructural parameters and the number of fragility fractures within the overall cohort. In line with previous reports, our exploratory findings suggest a potential association between impaired bone structure and fractures [41, 42]. Peripheral fragility fractures were particularly linked to deficits in trabecular bone density at both the distal radius and tibia. These exploratory findings highlight the potential contribution of trabecular compartment to fracture vulnerability, but should be interpreted cautiously given the limited sample size and cross-sectional design.

Besides its strengths, this study also has some limitations. Due to the limited sample size, the preliminary nature of the results must be considered. The limited cohort size also restricted the number of variables that could be included in the propensity score matching model beyond age and lowest T-score, so residual confounding by factors such as frailty- or sarcopenia-related parameters and detailed fall circumstances cannot be excluded. A structured fall history was not systematically available, which should be regarded as a limitation of the retrospective design. Notably, fractures attributable to high energy or adequate trauma were excluded a priori, limiting the cohort to low-trauma fragility fractures. Measurements were taken on the distal radius and distal tibia, thus no conclusions can be drawn about the exact bone microstructure at the proximal humerus. Moreover, the results should be interpreted with caution due to a potential selection bias, since only in cases of a fractured radius, the healthy contralateral side is measured. However, HR-pQCT sites are established surrogates for systemic skeletal microarchitecture. The exploratory association analyses were not powered for fracture subgroup comparisons and should therefore be interpreted with caution. The design of the retrospective cross-sectional study does not allow for causal conclusions. Accordingly, all associations reported in this study, including those between fracture type and HR-pQCT parameters, should be interpreted as cross-sectional associations rather than evidence of causal or temporal relationships. Nevertheless, inclusion of only postmenopausal, treatment-naïve women enhances internal validity. Furthermore, propensity score matching eliminated important confounding factors such as age and lowest T-score, thereby reducing bias. We additionally assessed overall comorbidity burden after propensity score matching using the CCI, which did not differ significantly between groups, nor did obesity prevalence, thereby reducing the likelihood of bias arising from differences in comorbidity between the two cohorts.

## Conclusion

PHF may be associated with a more pronounced cortical deficit characterized by reduced Ct.Th and unfavorable bone geometry with reduced cortical and trabecular area, not fully captured by aBMD. Nevertheless, it is important to investigate the mechanism of injury in addition to bone structure and geometry. Future studies with larger cohorts, longitudinal designs, and uniform imaging are needed to confirm these findings and further investigate their stratifying and prognostic significance.

## Supplementary Information

Below is the link to the electronic supplementary material.

Supplementary Material 1

## Data Availability

The relevant data is published in the manuscript. However, further data can be requested from the corresponding author upon reasonable request, but due to data protection regulations, restrictions may apply.
