# Supplementary material for: Distinct Cortical Microstructure in Postmenopausal Women with Distal Radius and Proximal Humerus Fractures
Source: Calcif Tissue Int. 2026 Jul 21;117(1):117. doi: 10.1007/s00223-026-01579-7 (PMC13388505; doi:10.1007/s00223-026-01579-7)
Supplement: Supplementary file 1 — Supplementary Material 1 [file 223_2026_1579_MOESM1_ESM.docx]

**Supplementary Material**

**Distinct cortical microstructure in postmenopausal women with distal radius and proximal humerus fractures**

Mikolaj Bartosik^1^, Alexander Simon^1^, Oskar Windels^1^, Felix N. von Brackel^1^, Florian Barvencik^1^, Michael Amling^1^, Ralf Oheim^1^

^1^ Department of Osteology and Biomechanics, University Medical Center Hamburg-Eppendorf, Hamburg, Germany

**Supplementary Table 1: Comparison of laboratory parameters between postmenopausal patients categorized by distal radius fracture (DRF) and proximal humerus fracture (PHF)**. ALP: alkaline phosphatase; 25-hydroxyvitamin D: 25(OH)D; b-ALP: bone specific alkaline phosphatase; C-terminal telopeptide of type I collagen: beta Crosslaps; PTH: parathyroid hormone; DPD/Crea, deoxypyridinoline per creatinine in the urine. Significant differences in the group comparisons are indicated by exact p-values and are marked in bold (p < 0.05). Effect sizes are given as Cohen's d, with medium effect sizes (Cohen's d > 0.5) highlighted by underlining.

|  |  |  | **DRF** | |  | **PHF** | |  |  |  |
| --- | --- | --- | --- | --- | --- | --- | --- | --- | --- | --- |
| Parameter | | Reference ranges | Mean | SD |  | Mean | SD |  | p-value | d |
| **Laboratory bone parameter** | |  |  |  |  |  |  |  |  |  |
|  | Calcium (mmol/l) | 2.18 - 2.60 | 2.46 | 0.10 |  | 2.41 | 0.13 |  | 0.110 | 0.43 |
|  | Phosphate (mmol/l) | 0.78 - 1.65 | 1.07 | 0.13 |  | 1.03 | 0.19 |  | 0.343 | 0.25 |
|  | ALP (U/l) | 46 - 116 | 83.5 | 23.5 |  | 87.2 | 29.9 |  | 0.725 | 0.10 |
|  | Osteocalcin (µg/l) | 5.4 - 59.1 | 25.5 | 5.5 |  | 22.8 | 8.2 |  | 0.140 | 0.39 |
|  | 25(OH)D (µg/l) | > 30.0 | 39.1 | 13.9 |  | 32.8 | 13.6 |  | **0.042** | 0.56 |
|  | b-ALP (µg/l) | 5.2 - 24.4 | 16.3 | 7.9 |  | 16.1 | 7.3 |  | 0.697 | 0.10 |
|  | Procollagen1-NT-Peptide (µg/l) | 22.0 - 116.0 | 88.0 | 35.0 |  | 78.3 | 34.7 |  | 0.368 | 0.27 |
|  | beta Crosslaps (µg/l) | 0.171 - 0.970 | 0.543 | 0.235 |  | 0.467 | 0.275 |  | 0.182 | 0.41 |
|  | PTH (ng/l) | 18.4 - 80.1 | 55.8 | 19.2 |  | 55.0 | 21.2 |  | 0.598 | 0.14 |
|  | DPD/Crea (nmol/mmol) | 3 - 7 | 9 | 3 |  | 9 | 2 |  | 0.835 | 0.06 |

**Supplementary Table 2: Comparison of muscle performance parameters.** CRT: chair rising test; EC: eyes closed; EO: eyes open; SD: standard deviation. Group comparisons are indicated by exact p-values. Effect sizes are given as Cohen's d, with medium effect sizes (Cohen's d > 0.5) highlighted by underlining.

|  |  |  | **DRF** | |  | **PHF** | |  |  |
| --- | --- | --- | --- | --- | --- | --- | --- | --- | --- |
| Parameter | | | Mean | SD |  | Mean | SD | p-value | d |
| **Mechanography** | | |  |  |  |  |  |  |  |
|  | Grip strength (kg) | | 19.0 | 7.6 |  | 16.0 | 8.6 | 0.224 | 0.45 |
|  | CRT time per repetition (s) | | 1.79 | 0.59 |  | 2.15 | 1.38 | 0.456 | 0.46 |
|  | Romberg path length EO (mm) | | 157.6 | 67.2 |  | 151.6 | 56.6 | >0.999 | <0.01 |
|  | Romberg path length EC (mm) | | 276.0 | 131.9 |  | 229.3 | 188.9 | 0.113 | 0.59 |
